# SOX11/PRDX2 axis modulates redox homeostasis and chemoresistance in aggressive mantle cell lymphoma

**DOI:** 10.1038/s41598-024-58216-2

**Published:** 2024-04-03

**Authors:** Anna De Bolòs, Marta Sureda-Gómez, Maria Carreras-Caballé, Marta-Leonor Rodríguez, Guillem Clot, Silvia Beà, Eva Giné, Elias Campo, Patricia Balsas, Virginia Amador

**Affiliations:** 1 Institut d’Investigacions Biomèdiques August Pi i Sunyer (IDIBAPS), Barcelona, Spain; 2 Centro de Investigación Biomédica en Red de Cáncer (CIBERONC), Madrid, Spain; 3 Department of Basic Clinical Practice, Faculty of Medicine, University of Barcelona, Barcelona, Spain; 4 Hematopathology Section, Pathology Department, Hospital Clínic Barcelona, Barcelona, Spain; 5 Hematology Department, Hospital Clínic, Barcelona, Spain

**Keywords:** Cancer, Cell biology, Molecular biology

## Abstract

Mantle cell lymphoma (MCL) is an incurable B-cell neoplasm characterized by an aggressive behavior, short responses to conventional therapies and SOX11 overexpression, which is associated with aggressive disease features and inferior clinical outcome of patients. Oxidative stress is known to induce tumorigenesis and tumor progression, whereas high expression levels of antioxidant genes have been associated with chemoresistance in different cancers. However, the role of oxidative stress in MCL pathogenesis and the involvement of SOX11 regulating redox homeostasis in MCL cells are largely unknown. Here, by integrating gene set enrichment analysis of two independent series of MCL, we observed that SOX11+ MCL had higher reactive oxygen species (ROS) levels compared to SOX11− MCL primary tumors and increased expression of *Peredoxine2* (*PRDX2*), which upregulation significantly correlated with SOX11 overexpression, higher ROS production and worse overall survival of patients. SOX11 knockout (SOX11KO) significantly reduced PRDX2 expression, and SOX11KO and PRDX2 knockdown (PRDX2KD) had increased ROS levels and ROS-mediated tumor cell death upon treatment with drugs, compared to control MCL cell lines. Our results suggest an aberrant redox homeostasis associated with chemoresistance in aggressive MCL through SOX11-mediated PRDX2 upregulation, highlighting PRDX2 as promising target for new therapeutic strategies to overcome chemoresistance in aggressive MCLs.

## Introduction

Mantle cell lymphoma (MCL) is one of the most aggressive non-Hodgkin lymphomas (NHL) characterized by its high dissemination to secondary lymphoid tissues, poor responses to standard chemotherapies and frequent relapses, with short median survival. MCL is still an incurable B-cell neoplasm, which needs advancement in the search for new oncologic signaling pathways to identify novel potential therapeutic targets. Interestingly, a subset of patients with a predominant leukemic non-nodal MCL (nnMCL) frequently have an indolent clinical evolution, even in absence of treatment^1,2^. SRY-related HMG-box gene 11 (SOX11) is aberrantly overexpressed in conventional MCL (cMCL), and negative or very weakly expressed in the nnMCL subtype^3–5^. Several studies have shown the role of SOX11, regulating different oncogenic mechanisms in the pathogenesis of MCL^6–11^ .

Deregulation of cellular energy is one of the hallmarks of cancer. Cancer cells have an altered metabolism, since they have increased energy requirements compared to normal cells^12^. Consequently, cancer cells produce high amounts of chemical sub-products, being reactive oxygen species (ROS) the most common one. Several studies have demonstrated the oncogenic role of ROS, promoting survival, proliferation, or angiogenesis^13^. To maintain cellular homeostasis, ROS levels are countered by antioxidants, such as superoxide dismutase, glutathione peroxidase, thioredoxin and peroxiredoxin. The role of these enzymes in cancer is complex, since they potentially prevent tumorigenesis by detoxification of ROS^14^, but on the other hand, they are associated with chemoresistance, eliminating lethal ROS-mediated cytotoxic response to chemotherapy^15^.

Oxidative stress plays an oncogenic role in the development and progression of several NHL^16^. Furthermore, overexpression of peroxiredoxins has been associated with chemoresistance in different lymphomas^17^. ROS-mediated cytotoxic response to bortezomib has been reported in MCL^18^. However, the mechanisms of ROS and antioxidant genes leading MCL pathogenesis are largely unknown. In the present study, we analyzed the mechanisms of cellular redox homeostasis and its association with chemoresistance in SOX11+ and SOX11− MCL.

## Results

### SOX11+ cases display enhanced oxidative stress gene signatures compared to SOX11− MCL cases

**Figure 1:**
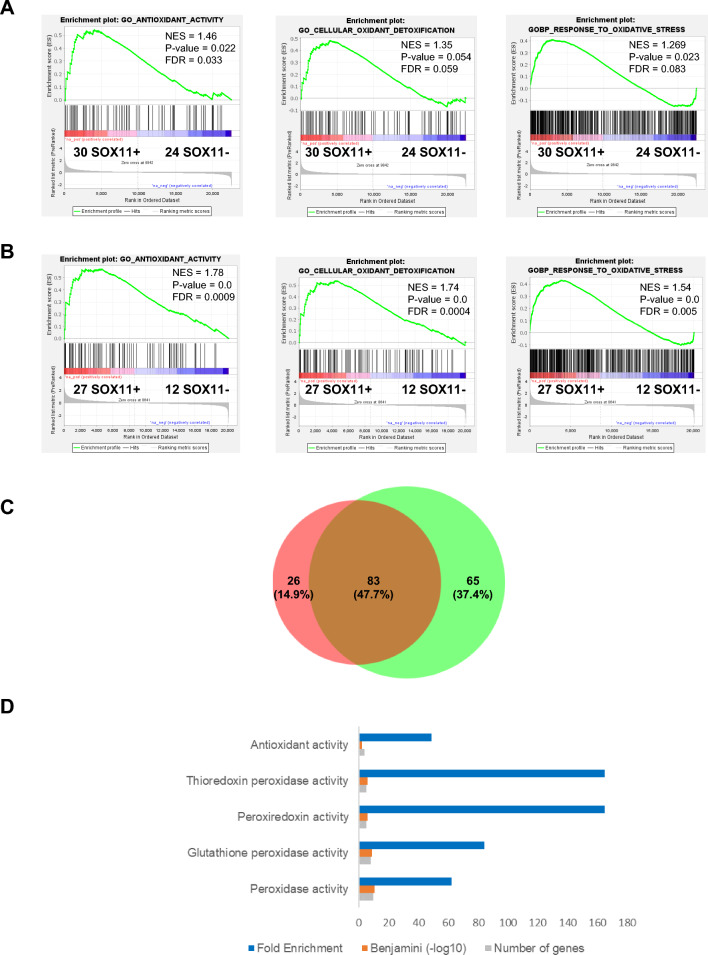
SOX11+ display enhanced oxidative stress gene signatures compared to SOX11− MCL cases. (A-B) GSEA analysis on GEP microarray data from two independent series of MCL primary samples (**A**) Series # 1 of PB samples from 30 SOX11+ and 24 SOX11− MCL cases and (**B**) Series # 2 of PB and LN from 27 SOX11+ and 12 SOX11− MCL cases, using oxidative stress-related gene signatures. Normalized enrichment score (NES), *p* value, and false-discovery rate (FDR) are shown. FDR < 0.1 indicates statistical significance. (**C**) Venn diagram showing the overlap between 109 enriched genes in SOX11+ vs. SOX11− MCL primary cases, using Series # 1 (orange circle) and 148 DEG using Series # 2 (green circle) (**D**) Molecular Function annotation analysis obtained by DAVID Software of 83 genes significantly upregulated in SOX11+ MCL primary cases in common in two independent series # 1 and # 2 of MCL cases. The most significant terms, number of genes, fold enrichment and adjust *p*-value (Benjamini) are indicated.

We first compared the expression of oxidative stress-related genes in SOX11+ vs. SOX11− MCL primary cases using previously published GEP data of two independent series of MCL: series # 1 composed of 54 peripheral blood (PB) MCL samples (30 SOX11+ and 24 SOX11-) (GSE79196)^19^ and series # 2 composed of 39 PB and LN samples (27 SOX11+ and 12 SOX11-) (EGAD00010001842)^20^. By GSEA, we observed that GEPs of SOX11+ MCL primary tumors were significantly enriched in antioxidant activity-, cellular oxidant detoxification- and response to oxidative stress-related gene set signatures compared to SOX11− tumors (Fig. 1A,B). Eighty-three (47.7%) of the oxidative stress-related genes enriched in SOX11+ cases overlapped between these two MCL series (Fig. 1C). Gene ontology (GO) analyses showed that glutathione peroxidase and peroxiredoxin were the molecular functions in which these 83 genes were most significantly involved (Supplemental Table 1 and Fig. 1D).

### PRDX2 upregulation correlates with SOX11 expression and associates with poor survival of patients with MCL

**Figure 2:**
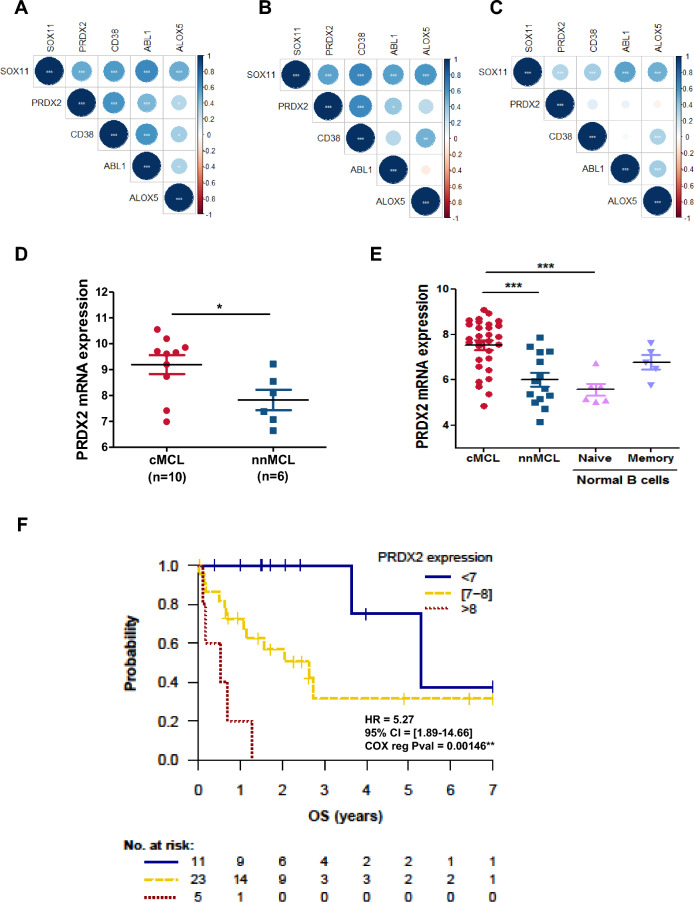
PRDX2 upregulation correlates with SOX11 expression and associates with poor survival of patients with MCL. (**A**–**C**) Correlation of SOX11 and PRDX2, CD38, ABL1 and ALOX5 mRNA levels in levels in (**A**) 30 SOX11+ and 24 SOX11− MCL cases, using series # 1; and (**B**) 27 SOX11+ and 12 SOX11− MCL cases, using series # 2 and (**C**) in 122 SOX11+ nodal MCL samples, using series # 3. Blue and red color indicates positive and negative correlation, respectively. Pearson statistical signification is indicated for each gene correlation. *p < 0.05, **p < 0.01, ***p < 0.001. (**D**) PRDX2 mRNA expression levels comparing GEP/RNA-seq data from series # 4 of 10 cMCL (SOX11 +) and 6 nnMCL (SOX11-) primary samples. (**E**) PRDX2 mRNA expression levels, comparing SOX11+ cMCL and SOX11− nnMCL of series # 4, with normal naïve and memory B cells of series # 5. The significance of difference was determined by independent samples Student t test: *p < 0.05, *** *p* < 0.001. (**F**) Kaplan–Meier curves and Cox regression analysis showing the association of PRDX2 mRNA levels with OS using GEP and clinical data of series # 1. The hazard ratio (HR) with 95% confidence interval (CI), the cox regression *p*-value, and the risk table (No. at risk) are shown.

We analyzed the correlation between the expression of SOX11 and the 83 oxidative stress-related genes enriched in SOX11+ tumors, using GEPs of MCL series # 1 and # 2 and another independent series # 3 of 122 LN samples, all of them from SOX11+ MCL primary tumors (GSE93291)^21^. We observed that only 4 of the 83 genes had a significant correlation between its upregulation and SOX11 overexpression in the three studied series (Supplemental Table 2, Fig. 2A–C, respectively).

As peroxiredoxin activity was one of the GO molecular functions with highest significant enriched in SOX11+ MCLs (Supplemental Table 1 and Fig. 1D), we decided to focus our further analyses to study the involvement of *Peroxiredoxin 2 (PRDX2*) gene in MCL oxidative stress. We validated that PRDX2 mRNA levels were significantly higher in SOX11+ cMCL compared to SOX11− nnMCL using GEP/RNA-seq data of Series # 4, composed of 10 SOX11+ and 6 SOX11− PB and LN primary MCL samples^22,23^ (Fig. 2D). Moreover, PRDX2 mRNA levels were significantly higher in cMCL compared to nnMCL and to naive and memory normal B-cells, its respective cells of origin (Series # 5; EGAS00001001197)^22^ (Fig. 2E). Interestingly, we did not observe correlation between the expression of PRDX2 and *TP53* alterations or expression in MCL cases (data not shown).

Next, we studied the prognostic impact of *PRDX2* expression in the MCL series # 1 and observed that patients expressing high PRDX2 mRNA levels had a significantly shorter OS (*p*-val = 0.001) (Fig. 2F). In a univariate Cox regression analysis, we observed an independent prognostic value of *PRDX2* gene expression when evaluated alongside two other MCL risk factors, the MCL proliferation signature and the number of copy number alterations (CNA) (Table 1). Interestingly, we analyzed PRDX2 mRNA levels in primary samples of responder’s and no-responder’s patients to chemotherapy in MCL series # 1 and # 2. We observed significantly higher PRDX2 mRNA levels in no-responder’s than in responder’s patients in the two independent series analyzed (Supplemental Figure S1), suggesting that PRDX2 might be involved in chemoresistance in MCL.

**Table 1:** Univariate Cox regression analysis used to evaluate the independent prognostic value of PRDX2 mRNA expression.

	HR	95% CI	*p*-value
PRDX2 mRNA expression	5.601	[1.93–16.27]	0.0015
Log (CNA+ 1)	1.629	[1.09–2.42]	0.0152
Proliferation (scaled)	3.623	[1.55–8.44]	0.0028

Two other high-risk factors were considered, the number of copy number alterations (CNA, in logarithmic scale), and an MCL proliferation signature (scaled). This analysis was performed with the MCL primary series # 1.

N = 38, number of events = 18.

Together, these results suggest that PRDX2 upregulation correlates with SOX11 overexpression and associates with worse outcome of the patients, suggesting that PRDX2 could be involved in MCL pathogenesis.

### PRDX2 upregulation correlates with increased ROS levels in MCL primary tumors

**Figure 3:**
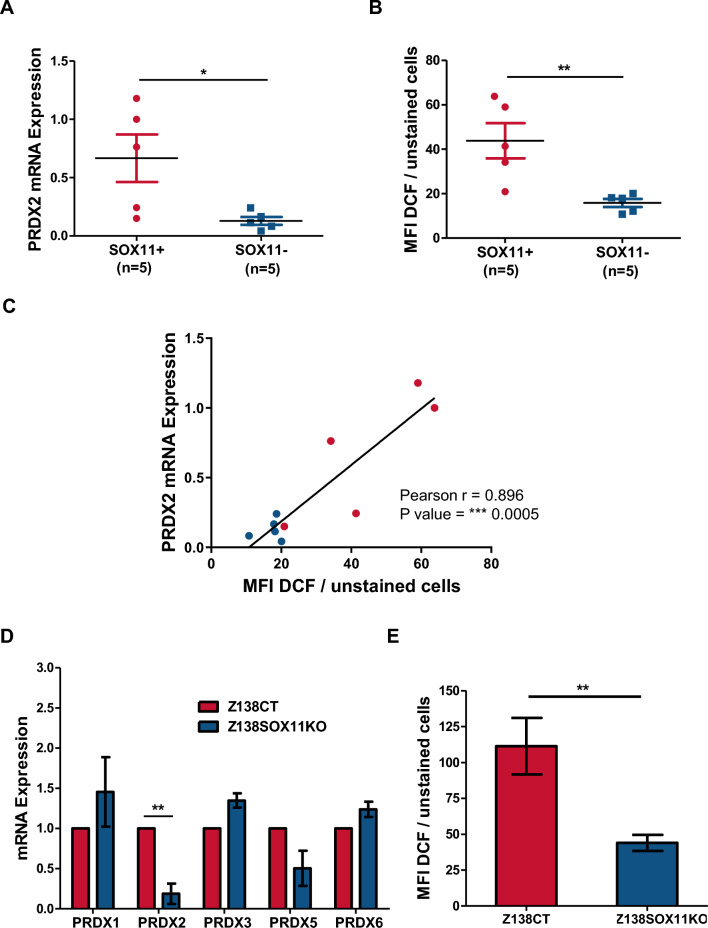
PRDX2 upregulation correlates with increased ROS levels in MCL cases. (**A**) PRDX2 mRNA expression levels, obtained by qPCR relative to GUSB mRNA levels, and (**B**) Graph showing the median fluorescence intensity (MFI) of DCF dye, in 5 SOX11+ and 5 SOX11− MCL primary samples. Results are relative to unstained cells. (**C**) Graph showing the correlation between ROS production (MFI of DCF dye) and PRDX2 mRNA expression levels, in 5 SOX11+ (red) and 5 SOX11− (blue) MCL primary samples. Pearson correlation coefficient and *p* value are shown. (**D**) PRDXs 1–3, 5, 6 mRNA expression levels, analyzed by RTqPCR relative to GUS mRNA levels, in Z138CT compared to Z138SOX11KO MCL cell lines. Results are relative to Z138CT for each gene. (**E**) Median fluorescence intensity of DCF dye in Z138CT and Z138SOX11KO MCL cell lines. Results are relative to unstained cells and Z138CT. Bar plot represents the mean percentage ± standard deviation of 3 independent experiments. The significance of difference was determined by independent samples Student t test: *p < 0.05, **p < 0.01.

Interestingly, we confirmed the higher PRDX2 mRNA levels analyzed by qPCR in 10 purified primary MCL samples (5 SOX11+ and 5 SOX11-) (Fig. 3A). We also observed significantly higher basal levels of ROS production in these SOX11+ compared to SOX11− MCL primary samples (Fig. 3B). In line with the crucial function of peroxiredoxins neutralizing ROS generated by oxidative stress in tumor cells^24,25^, we observed a significant positive correlation between ROS and PRDX2 mRNA levels, but not with other PRDXs (data not shown), in these MCL primary tumors (Fig. 3C).

The relationship between SOX11 expression, PRDX2 and ROS production was confirmed experimentally in the SOX11+ Z138 MCL cell lines. Z138 SOX11 knockout cells (Z138SOX11KO) showed significant lower mRNA levels of PRDX2, but not of other PRDXs, (Fig. 3D) and ROS production (Fig. 3E) than control (Z138CT) cells. Furthermore, the SOX11-negative MCL cell line, JVM2, ectopically overexpressing SOX11 (JVM2SOX11 +) exhibited higher PRDX2 mRNA levels and ROS production than its control MCL cell line (JVM2CT) (Figure S2A-B, respectively). All these observations suggest that SOX11 could be involved in redox homeostasis through the upregulation of PRDX2 to counter ROS levels in MCL.

### Hypoxia induces ROS production in SOX11+ MCL cells

**Figure 4:**
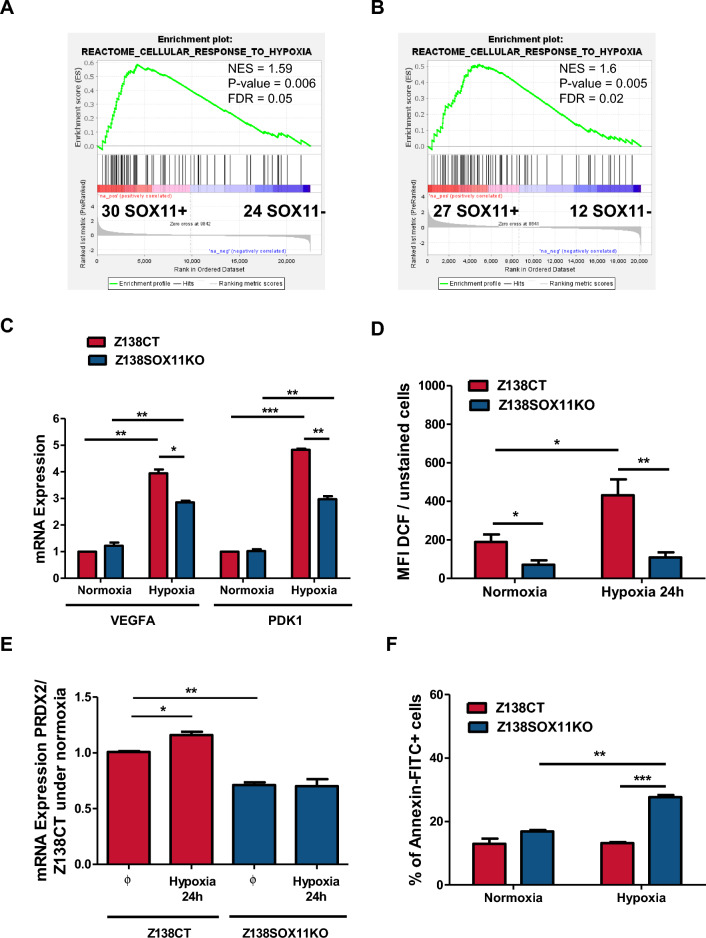
A hypoxic cell microenvironment induces ROS production, and PRDX2 expression in SOX11+ MCLs. (**A**,**B**) GSEA analysis on GEP microarray data from two independent series of MCL primary samples (**A**) series #1 of 30 SOX11+ cMCL and 24 SOX11− nnMCL cases and (**B**) series #2 of 27 SOX11+ and 12 SOX11− MCL cases, using response to hypoxia-related signatures. Normalized enrichment score (NES), *p* value, and false-discovery rate (FDR) are shown. FDR < 0.1 indicates statistical significance. (**C**) VEGFA and PDK1 mRNA expression levels in Z138CT and Z138SOX11KO MCL cell lines growing under normoxic and hypoxic (1.2% O_2_) conditions for 24 h (h). Results refer to Z138CT cells in normoxia. (**D**) MFI of DCF dye in Z138CT and Z138SOX11KO cells growing in hypoxic compared to normoxic (1.2% O_2_) conditions for 24 h. Results are relative to unstained cells. (**E**) PRDX2 mRNA in Z138CT and Z138SOX11KO MCL cell lines in normoxic (Ø) and hypoxic (1.2% O_2_) conditions, for 24 h. Results are refereed to Z138CT cells in normoxia. (**F**) % of Annexin-FITC+ cells in Z138CT and Z138SOX11KO cells under normoxic and hypoxic (1.2% O_2_) conditions for 24 h. The significance of difference was determined by independent samples Student t test: *p < 0.05, **p < 0.01, ***p < 0.001.

GSEA showed that SOX11+ primary tumors, in series # 1 and # 2, also were enriched in hypoxia-mediated cellular response gene signatures, compared to SOX11− MCL cases (Fig. 4A,B, respectively). A hypoxic microenvironment is a recognized ROS-inducer factor^26^. Since we observed that SOX11+ cells were producing higher levels of ROS than SOX11− MCL cells (Fig. 3B,E), we wondered if a hypoxic cell microenvironment would induce ROS production in aggressive MCLs. To confirm this idea, we cultured MCL cell lines under hypoxic conditions. The levels of two well-known hypoxia-induced genes, *VEGFA* and *PDK1*^27,28^, were significantly increased in MCL cell lines in hypoxia compared to normoxia, but this increase was significant higher in Z138CT than in Z138SOX11KO cells (Fig. 4C). Interestingly, the levels of ROS production and PRDX2 mRNA levels significantly increased in Z138CT, but not in Z138SOX11KO cells growing under hypoxic compared to normoxic conditions (Fig. 4D,E, respectively). Similar results were observed in JVM2SOX11+ compared to JVM2CT cells (Supplemental Figure S2A-B, respectively). However, cell death in vitro experiments showed that Z138SOX11KO had a significant lower survival than Z138CT cells in hypoxia compared to normoxia (Fig. 4F).

Together, our results demonstrated the contribution of hypoxia to ROS production in MCL, as previously shown in other aggressive tumors^29^, and suggest that SOX11 might be involved in cell adaptation to hypoxia via increased levels of PRDX2 and ROS in MCL cells.

### PRDX2 promotes chemoresistance by regulating ROS levels in MCL cells

**Figure 5:**
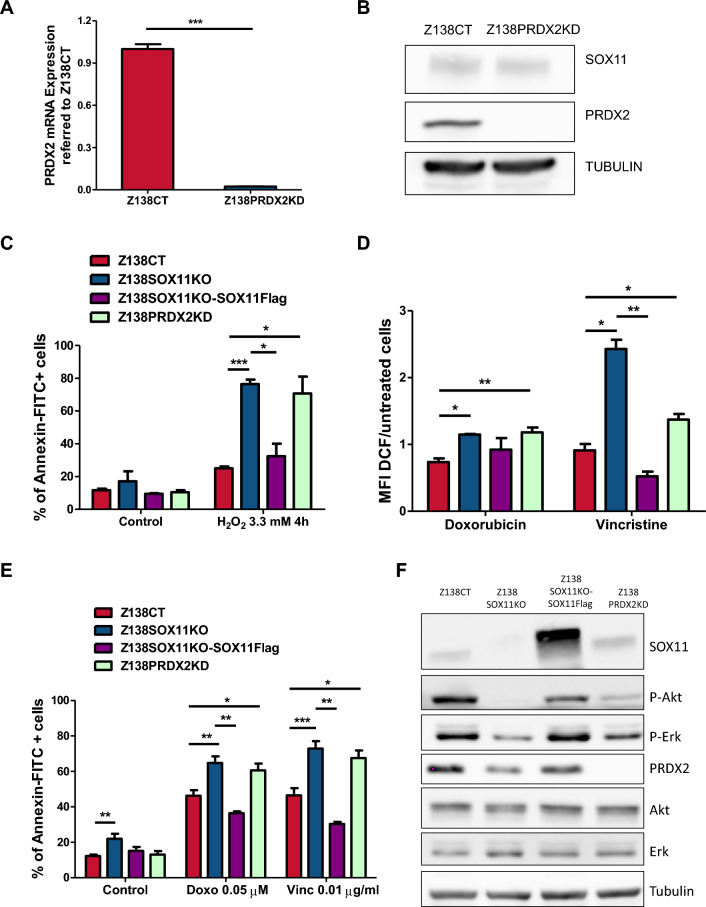
SOX11-mediated PRDX2 upregulation promotes chemoresistance by regulating ROS levels in MCL. (**A**) PRDX2 mRNA expression levels, obtained by RTqPCR relative to GUSB mRNA levels, in Z138CT and Z138PRDX2KD MCL cell lines. Results are relative to Z138CT. (**B**) WB analysis showing SOX11 and PRDX2 protein levels in Z138CT and Z138PRDX2KD MCL cell lines. (Full-length blots corresponding to cropped blots are shown in Supplemental Figure S4). (**C**) % of Annexin-FITC+ cells in Z138CT, Z138SOX11KO, Z138SOX11KO-SOX11Flag and Z138PRDX2KD MCL cell lines after treated with 3.3 mM hydrogen peroxide (H_2_O_2_) for 4 h in culture under hypoxia conditions (1.2% O_2_) for 24 h. (**D**) MFI of DCF dye in Z138CT, Z138SOX11KO, Z138SOX11KO-SOX11Flag and Z138PRDX2KD MCL cells lines after 0.05 µM doxorubicin or 0.01 µg/ml vincristine treatment, under hypoxic conditions (1.2% O_2_) for 24 h. Results are related to unstained and untreated cells. (**E**) % of Annexin-FITC+ in Z138CT, Z138SOX11KO, Z138SOX11KO-SOX11Flag and Z138PRDX2KD MCL cells lines in untreated cells (Control) and after the treatment with 0.05 µM doxorubicin (Doxo) and 0.01 µg/ml vincristine (Vinc) under hypoxic conditions (1.2%O_2_) for 24 h. The significance of difference was determined by independent samples Student t test: *p < 0.05, **p < 0.01, ***p < 0.001. (**F**) WB analysis showing SOX11, PRDX2, P-AKT, P-ERK, AKT and ERK protein levels in Z138CT, Z138SOX11KO, Z138SOX11KO-SOX11Flag and Z138PRDX2KD MCL cells lines under hypoxia conditions (1.2%O_2_) for 24 h. Tubulin was used as loading control. (Full-length blots corresponding to cropped blots are shown in Supplemental Figure S5).

To elucidate the role of PRDX2 in MCL, we silenced *PRDX2* gene in Z138CT cells, by stable transduction with specific shRNAs lentiviral vector, generating a newly Z138PRDX2 knockdown (KD) MCL cell line, with reduced PRDX2 mRNA (Fig. 5A) and protein levels (Fig. 5B and Supplemental Figure S4).

To analyze if PRDX2 was involved in oxidative stress, we performed in vitro cell death experiments to evaluate the response of our MCL cell line models to the hydrogen peroxide (H_2_O_2_) oxidant agent. We observed a significant increase in apoptosis in Z138SOX11KO and Z138PRDX2KD cell lines upon treatment with 3.3 mM of H_2_O_2_ for 4 h, under hypoxic conditions, compared to Z138CT cells. Interestingly, cell viability was rescued upon SOX11 ectopic overexpression in Z138SOX11KO (Z138SOX11KO-SOX11Flag) cells, reaching similar levels of survival than Z138CT cell line (Fig. 5C).

To analyze if PRDX2 was involved in drug resistance, as reported for other tumors^24,30,31^, we next evaluated the response of our MCL cell line models to drugs strongly dependent on ROS-induced cytotoxicity, like doxorubicin and vincristine. These two compounds are part of CHOP chemotherapy, a standard element used in first line treatment for MCL. Interestingly, we observed that ROS levels significantly increased in Z138SOX11KO and Z138PRDX2KD cell lines compared to Z138CT cells, after treatment with doxorubicin and vincristine (Fig. 5D). Moreover, in vitro experiments showed that Z138SOX11KO and Z138PRDX2KD MCL cell lines displayed a significantly higher sensitivity to both drugs (doxorubicin and vincristine), showing a significant higher number of apoptotic cells than Z138CT cells (Fig. 5E). Furthermore, ROS production and cell viability was rescued in Z138SOX11KO-SOX11Flag, reaching similar ROS levels and drug resistance to the ones observed in Z138CT cells (Fig. 5D,E, respectively).

Remarkable, pre-incubation of MCL cell lines with the antioxidant agent N-acetyl-cysteine (NAC) significantly reduced doxorubicin- and vincristine-induced cell apoptosis in Z138SOX11KO and showed a tendency in Z138shPRDX2 cells compared to Z138CT cell lines. Interestingly, NAC pretreatments did not show any effect on Z138SOX11KO cell line ectopically overexpressing SOX11 (Z138SOX11KO-SOX11Flag) (Supplemental Figure S3).

ROS plays an oncogenic role by contributing to the activation of oncogenic signaling pathways, including AKT and ERK pathways^32^. Here, under hypoxia conditions, we observed that p-AKT and p-ERK protein levels, but not their non-phosphorylated forms, decreased not only in Z138SOX11KO cells, as previously described upon SOX11 silencing^9^, but also in Z138PRDX2KD compared to Z138CT MCL cell lines. Interestingly, PRDX2, as well as p-AKT and p-ERK protein levels were rescued in Z138SOX11KO-SOX11Flag, reaching similar protein levels as in Z138CT cells (Fig. 5F and Supplemental Figure S5).

These results suggest a key role of SOX11-mediated PRDX2 upregulation, contributing to chemoresistance by neutralizing lethal ROS levels and probably activating oncogenic signaling pathways in MCL cells.

## Discussion

Oxidative stress caused by the imbalance between ROS and antioxidant system has been largely associated with diverse pathologies, including malignant neoplasms^33^. Furthermore, several drugs used to treat cancers are highly dependent on ROS-mediated cytotoxicity^34^. Therefore, expanding our knowledge about tumor cell redox homeostasis in response to treatment could be very useful to decipher cancer pathogenesis and overcome drug resistance. This is particularly interesting for MCL, an incurable disease with very short median survival and frequent cases with relapsed/refractory disease upon first line chemotherapy. In line with previous publications describing association between cellular redox environment and cancer, including aggressive lymphomas^35^, we observed the upregulation of oxidative stress-related genes and a significantly higher production of ROS in aggressive SOX11+ cMCLs compared to SOX11− nnMCL cases. We identified *PRDX2* as one of the most significant upregulated antioxidant genes, within the oxidative stress-related gene set signatures, which significantly correlated with SOX11 overexpression in MCL cells. High levels of antioxidant proteins have been observed in different cancer cells associated with increased tumor cell survival through maintenance of cellular redox balance, under persistent endogenous oxidative stress^36^. In line, we observed a significant correlation between PRDX2 upregulation and increased ROS levels in primary MCL cells, and a significant association between higher levels of PRDX2 and worse overall survival of the patients and chemoresistance. *PRDX2* gene expression has been associated with several human malignancies, including B-cell lymphomas^37,38^. PRDX2 upregulation had prognostic value independently of common high-risk features in MCL, like the proliferation signature and CNA.

Hypoxia is a hallmark of tumor microenvironment in most growing tumors^39^. Diminished oxygen disposal contributes to tumors heterogeneity and promotes a stronger aggressive neoplasm phenotype^40^. HIF-1α expression is significantly increased in MCL and associates with a more aggressive disease^41^. In line, we observed a significant upregulation of cellular response-related genes to hypoxia in SOX11+ compared to SOX11− MCL.

Previous publications have shown that hypoxia increases ROS levels in tumors cells^42^. Increase in hypoxia-mediated ROS levels is an adaptive mechanism associated with tumor progression and aggressiveness^43^. In agreement, we observed that ROS production, and PRDX2 mRNA levels increased under hypoxic conditions only in SOX11+ MCLs. On the contrary, apoptosis significantly increased only in SOX11− cells growing under hypoxic compared to normoxic conditions, suggesting that SOX11 might be involved in cell adaptation to hypoxia via ROS production and increased PRDX2 levels in MCL cells.

Adaptation to high oxidative stress is an essential mechanism for tumors to survive and progress^44^. In MCL, the cellular mechanisms for oxidative stress adaptation have been studied only in response to bortezomib treatments, showing that it is mediated by ROS-induced cytotoxicity^18,45^. However, the mechanisms of ROS and antioxidant genes leading MCL pathogenesis and progression are largely unknown. Here, we extended our knowledge by analyzing oxidative stress in in vitro MCL cell line models, treating MCL cells with doxorubicin and vincristine (components of the CHOP chemotherapy), under hypoxic conditions. We observed a significant increase in ROS production and apoptosis in Z138SOX11KO and Z138PRDX2KD MCL cell lines compared to Z138CT cells. Also, ROS depletion using NAC reduced cell death induced by drugs in Z138SOX11KO and Z138PRDX2KD MCL cell lines. These results suggest that SOX11 and PRDX2 might be crucial to maintain redox balance, by countering lethal ROS levels after chemotherapy, to guarantee tumor cell survival in MCL. Interestingly, cell survival was rescued upon SOX11 ectopic overexpression in Z138SOX11KO, reaching similar levels as Z138CT cells. Together, these results suggest that SOX11-mediated PRDX2 upregulation could contribute to drug resistance in MCL through the regulation of ROS homeostasis, as described for other tumors^46^. We also observed that signaling pathways activated by ROS production were reduced in Z138SOX11KO and Z138PRDX2KO MCL cell lines, displaying lower p-AKT and p-ERK protein levels, compared to Z138CT cells.

**Figure 6:**
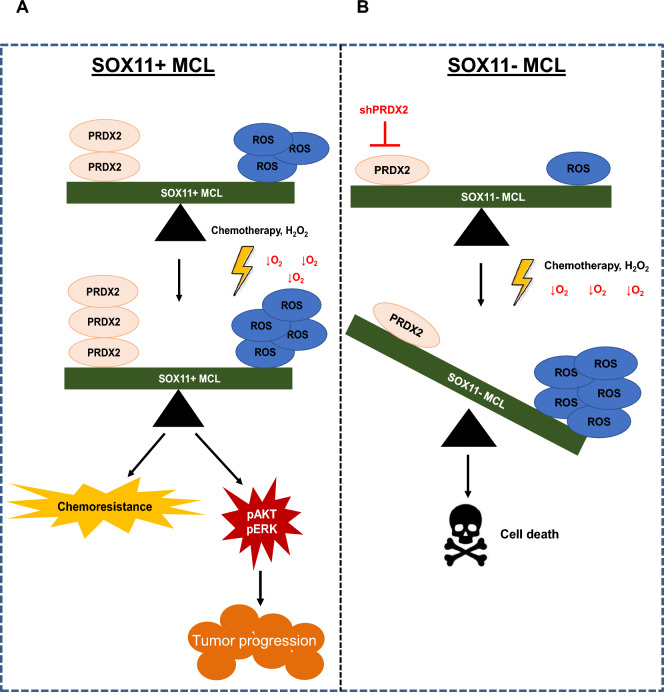
Hypothetical model for SOX11 and PRDX2 regulating oxidative stress response in MCL. (**A**) SOX11-mediated PRDX2 upregulation under chemotherapy-mediated oxidative stress caters ROS levels and promotes chemoresistance, tumor cell survival and oncogenic pathway activation in SOX11+ MCLs. (**B**) The low levels of PRDX2 in SOX11− MCL cases, SOX11KO and PRDX2KD MCL cell lines is not able to manage oxidative stress generated during hypoxia and/or chemotherapy, producing lethal levels of ROS that promotes tumor cell death in MCL.

Together, our results suggest a model in which SOX11+ MCL cells survive under hypoxic conditions through the modulation of ROS levels, as an adaptive mechanism. SOX11-mediated PRDX2 upregulation is crucial for ROS detoxification in MCL cell. Moreover, PRDX2 upregulation allow cells to survive to additional oxidative stresses generated during chemotherapy in aggressive SOX11+ MCL cells. PRDX2 may counteract ROS lethal levels but, on the contrary, may promote ROS-mediated oncogenic pathway activation in MCL (Fig. 6A). However, in SOX11− MCL cells, the low levels of PRDX2 are not able to manage oxidative stress generated during chemotherapy, and eventually tumor cells die upon treatments due to the increased lethal levels of ROS in SOX11− MCLs cells. SOX11KO or PRDX2 silencing significantly unbalance tumor redox homeostasis, becoming tumor cells very sensitive to chemotherapy due to the lethal levels of ROS produced in MCL cells (Fig. 6B).

Overall, our results suggest that SOX11 through PRDX2 might be crucial for tumor cells in the adaptation to hypoxia and oxidative stress, protecting them from drug-mediated cell death through the modulation of ROS lethal levels and its mediated cytotoxic response, promoting oncogenic pathway activation in MCL. Our results suggest a mechanistic pathway explaining oxidative stress-mediated chemoresistance in aggressive MCL. Targeting PRDX2 could be a promising therapeutic alternative for patients with chemoresistant/refractory MCLs.

## Methods

### Mantle cell lymphoma cell line models and primary tumors

The well characterized SOX11+ MCL cell line Z138 (CRL-3001; ATCC) was used to generate the stable transduced PRDX2 knockdown cell lines (Z138PRDX2KD), using shRNA lentiviral vectors (pLV-Puro-U6shPRDX2, from Vector Builder). Z138CT, Z138PRDX2KD, Z138SOX11-knockout (KO)^47^, Z138SOX11KO-SOX11Flag^47^, JVM2SOX11+ and JVM2CT^9^ MCL cell lines were used for in vitro experiments. HEK-293 T cell line (ATCC CRL-3216) was used for lentivirus production.

Cells were cultured at 37 ºC and 5% CO_2_ in RPMI 1640 medium with L-glutamine (Sigma-Aldrich, St Louis, MO) (Z138, Z138PRDX2KD, Z138SOX11KO and Z138SOX11KO-SOX11Flag) or DMEM with L-glutamine (Lonza) (HEK-293 T) supplemented with 10% fetal bovine serum (FBS; Sigma), 100 µg/ml streptomycin and 100 U/ml penicillin (Invitrogen).

Highly purified tumor cells (95%) from 10 primary MCLs (5 SOX11+ and 5 SOX11-) were used for in vitro experiments. MCL cells were isolated by Ficoll-Hypaque density gradient centrifugation (GE Healthcare) and cryopreserved in the Hospital Clínic/IDIBAPS tumor Biobank (Barcelona, Spain). For in vitro experiments, MCL primary cells were thawed and cultured at 37 ºC and 5% CO_2_ in RPMI 1640 supplemented with 10% FBS, 100 µg/ml streptomycin and 100 U/ml penicillin.

All cases had the t(11;14) and/or cyclin D1 expression^48^. MCL cells from MCL primary tumors were cultured in RPMI complete medium. All these tumors were diagnosed according to previously defined criteria^4,48^. The study was approved by the Institutional Review Board of the Hospital Clínic, Barcelona, Spain (Hospital Clínic de Barcelona Ethics Institutional Review Board). Written informed consent was obtained from all participants and the Ethics Committee approved this consent procedure. All methods were performed in accordance with the relevant guidelines and regulations by including a statement.

### Dataset analysis

For gene set enrichment analysis (GSEA), differential gene expression (DGE) analysis, correlation of different parameters, survival and/or multivariate COX regression analysis, we used four independent previously published microarray or RNA-sequencing (RNA-seq) gene expression profile (GEP) datasets and clinical data of samples from patients with MCL (GSE79196, EGAD00010001842, GSE93291, EGAD00001009422) and RNA-seq GEP datasets of different subtypes of normal B-cells (EGAS00001001197):

Series #1: Composed of 54 purified (> 95%) leukemic MCL cells (30 SOX11+ cMCL and 24 SOX11− nnMCL) (GSE79196)^19^. Survival analysis was performed with 39 patients that had clinical information, after the elimination of patients whose samples were obtained post-treatment or were treated with allotransplant. For multivariate COX regression analysis, we computed a proliferative score using the 17 genes previously described in (22), where the expression of the 13 overexpressed genes was added and the expression of the 4 under-expressed ones was subtracted.

Series #2: Composed of 27 SOX11+ and 12 SOX11− peripheral blood (PB) and lymph node (LN) samples from MCL primary tumors, after removing 5 cases, one case from skin and four cases that overlapped with series #1 (EGAD00010001842)^20^.

Series # 3: Composed of 122 SOX11+ nodal MCL (GSE93291)^21^. One case was considered as SOX11− MCL, based on the levels defined in our previous studies^19,49^, and removed from all the analyses performed with this dataset.

Series # 4: Composed of 12 purified leukemic and LN tumor cells from MCL patients of both previous cohorts (8 SOX11+ and 4 SOX11-), previously published (EGAD00001009422)^47^. We integrated this information with previously published RNA-seq data from 2 SOX11+ and 2 SOX11− MCL primary cases (purified PB tumor cell samples) obtained from the BLUEPRINT consortium^22,23^.

Series # 5: Composed of different subtypes of normal B cells, including naive B-cells (6 from PB and 4 from tonsil) and memory B-cells (5 samples) (EGAS00001001197)^50^. These data were normalized with samples from EGAD00010001842 series to compare the expression of MCL cells with normal B-cells, as previously described^47^.

### GSEA analyses

For GSEA analyses, oxidative stress-related gene sets were used in GEP data. GSEA was performed on preprocessed microarray data by fRMA with GSEA v4.3.2 using pre-ranked list of genes. For the analysis, data was randomized by 1000 permutations phenotype. Larger sets (> 500) were excluded. Gene sets were considered as differently expressed between SOX11+ vs SOX11− MCL primary cases at *p*-value < 0.05 and/or FDR < 0.2. The lists of genes obtained from GSEA were used for functional annotation analysis using DAVID Software (https://david.ncifcrf.gov/tools.jsp). For correlation analysis, we used the average of all probes for each gene.

### RNA extraction, cDNA generation and RT-qPCR

RNA extraction from MCL lines was performed using the RNeasy® Plus Mini Kit according to the manufacturer´s protocol instructions (Qiagen). 500 ng of RNA were used to generate cDNA using the qScript™ cDNA Synthesis Kit according to the manufacturer´s protocol instructions (Quanta Bioscience). cDNA was used to analyze mRNA expression levels by RT-qPCR using Fast SYBR Green Master Mix (Applied Biosystems) and specific primers (Supplemental Table 3), following manufacturers recommendations. GUSB was used as normalization control.

### Western blot analysis

Protein extract preparation and western blot analysis were performed as previously described^47^. Primary antibodies used were: PRDX2 (ab10367; Abcam), SOX11 (MRQ-58; Cell Marque), p-AKTS473 (Cell Signaling), AKT (sc-1618; Santa Cruz Biotechnology), p-MAPK T202/Y204 (p-ERK 42/44; Cell Signaling), ERK1/2 (sc-94; Santa Cruz Biotechnology) and α-tubulin (Abcam).

### Intracellular ROS production and cell death assays

ROS production in MCL cells was determined using the fluorescent probe H2DCFDA (Thermo Fisher). Briefly, 250 000 cells, grown in 24-well plates, were incubated with or without 10 µM of H2DCFDA in culture medium for 30 min at 37ºC. Then, cells were washed with PBS twice and re-suspended in PBS. The presence of ROS converted the non-fluorescent H2DCFDA into the highly fluorescent compound 2',7'-dichlorofluorescein (DCF), which was detected by flow cytometry in a FACSCanto 3 Laser cytometer (BD biosciences). Results were relative to unstained cells. To study ROS depletion, cells were preincubated with N-acetyl-cysteine (NAC) at 2 mM for 1 h before drugs treatment. To analyze cell death, MCL cells untreated or after incubation under hypoxic conditions (1.2% O_2_ for 24 h) with 0.05 µM doxorubicin or 0.01 µg/ml vincristine for 24 h or 3 mM H_2_O_2_ for 4 h, were labeled with Annexin- FITC (eBioscience) and analyzed by flow cytometry.

### Statistics

An unpaired two-tailed Student t-test was used to compare continuous variables between groups. Data are represented as mean ± standard deviation of 3 independent experiments and results were considered statistically significant when *p* < 0.05. Pearson correlation was used to measure the association between continuous variables. The clinical endpoint was overall survival (OS) calculated at time of sampling. Survival curves were estimated using the Kaplan–Meier method. Cox regression was used to evaluate the association between OS and continuous variables, to evaluate the independent prognostic value of multiple variables, and to estimate hazard ratios. *p*-values were adjusted for multiple testing using the Benjamini–Hochberg method. Statistical tests were performed using the R statistical software (version 3.6.9) or GraphPad Prism 5 software.

### Supplementary Information

Supplementary Information.

## Data Availability

All data generated or analyzed during this study are included in this published article.
