# Author Correction: SOX11/PRDX2 axis modulates redox homeostasis and chemoresistance in aggressive mantle cell lymphoma

**DOI:** 10.1038/s41598-024-61080-9

**Published:** 2024-06-11

**Authors:** Anna De Bolòs, Marta Sureda-Gómez, Maria Carreras-Caballé, Marta-Leonor Rodríguez, Guillem Clot, Silvia Beà, Eva Giné, Elias Campo, Patricia Balsas, Virginia Amador

**Affiliations:** 1 Institut d’Investigacions Biomèdiques August Pi i Sunyer (IDIBAPS), Barcelona, Spain; 2 Centro de Investigación Biomédica en Red de Cáncer (CIBERONC), Madrid, Spain; 3 Department of Basic Clinical Practice, Faculty of Medicine, University of Barcelona, Barcelona, Spain; 4 Hematopathology Section, Pathology Department, Hospital Clínic Barcelona, Barcelona, Spain; 5 Hematology Department, Hospital Clínic, Barcelona, Spain

Correction to: *Scientific Reports* 10.1038/s41598-024-58216-2, published online 03 April 2024

The original version of this Article contained errors in the Affiliations.

Affiliation 1 was incorrectly given as ‘Centre Esther Koplowitz (CEK), Institut d’Investigacions Biomèdiques August Pi i Sunyer (IDIBAPS), C/ Rosselló 149-153, 08036 Barcelona, Spain.’ The correction affiliation is listed below.

Institut d'Investigacions Biomèdiques August Pi i Sunyer (IDIBAPS), Barcelona, Spain.

Affiliation 3 was incorrectly given as ‘University of Barcelona, Barcelona, Spain.’ The correction affiliation is listed below.

Department of Basic Clinical Practice, Faculty of Medicine University of Barcelona, Barcelona, Spain.

In addition, the original version of this Article omitted an affiliation for Anna De Bolòs. The correct affiliations are listed below.

Institut d'Investigacions Biomèdiques August Pi i Sunyer (IDIBAPS), Barcelona, Spain.

Centro de Investigación Biomédica en Red de Cáncer (CIBERONC), Madrid, Spain.

Eva Giné was incorrectly affiliated with ‘Centro de Investigación Biomédica en Red de Cáncer (CIBERONC), Madrid, Spain’ and ‘University of Barcelona, Barcelona, Spain’.

The correct affiliations are listed below.

Institut d'Investigacions Biomèdiques August Pi i Sunyer (IDIBAPS), Barcelona, Spain.

Hematology Department Hospital Clínic Barcelona Spain.

The original Article has been corrected.

