# Supplementary material for: SOX11/PRDX2 axis modulates redox homeostasis and chemoresistance in aggressive mantle cell lymphoma
Source: Sci Rep. 2024 Apr 3;14:7863. doi: 10.1038/s41598-024-58216-2 (PMC10991377; doi:10.1038/s41598-024-58216-2)
Supplement: Supplementary file 1 — Supplementary Information. [file 41598_2024_58216_MOESM1_ESM.pdf]

SUPPLEMENTAL DATA

SOX11/PRDX2 AXIS MODULATES REDOX HOMEOSTASIS AND CHEMORESISTANCE IN AGGRESSIVE MANTLE CELL LYMPHOMA

Anna De Bolòs[1†], Marta Sureda-Gómez[1†], Maria Carreras-Caballé[1†], Marta-Leonor Rodríguez[1], Guillem Clot[1,2,3], Silvia Beà[1,2,3,4], Eva Giné[2,3], Elias Campo[1,2,3,4], Patricia Balsas[1††], Virginia Amador[1,2††*]

Supplemental Table S1…………………………………………..………........... Page 2

Supplemental Table S2……………………………………...…………….....Page 3-7

Supplemental Table S3……………... Page 8

Supplemental Figures S1……………………………...…… ……… ....….Pages 9

Supplemental Figure S2………………………………………………………...Page 10

Supplemental Figure S3 ………………………………………………….......Page 11

Supplemental Figure S4 ……………………………...………..……………....Page 12

Supplemental Figure S5 ……………………………………..…………...…Page 13-14

SUPPLEMENTAL TABLES

Table S1. GO annotations for molecular functions using the 83 significant DEG in SOX11+ vs SOX11- MCLs in two MCL primary series # 1 **and series** # 2**.** Only GO terms with Benjamini values <0.05 are shown.

GO term	p-value	Benjamini-value
Peroxidase activity	4.3E-14	1.6E-11
Glutathione peroxidase activity	3.9E-12	7.3E-10
**Peroxiredoxin activity**	1.2E-8	1.1E-6
Thioredoxin peroxidase activity	1.2E-8	1.1E-6
Identical protein binding	3.4E-5	2.5E-3
**Antioxidant activity**	7.5E-5	4.7E-3
Glutathione transferase activity	2.2E-4	1.2E-2
Enzyme binding	2.2E-4	1.2E-2
Protein binding	6.5E-4	2.7E-2
RNA polymerase II sequence-specific DNA binding transcription factor binding	1.2E-3	4.4E-2
Protein homodimerization activity	1.3E-3	4.4E-2
Protein C-terminus binding	1.5E-3	4.8E-2

Table S2. Correlation between the expression of SOX11 and the 83 oxidative stress- and antioxidant-related genes commonly enriched in SOX11+ compared to SOX11- MCL samples in GEPs of the MCL series # 1, # 2 and # 3 (GSE79196, EGAD00010001842 and GSE93291, respectively). R Pearson coefficient and p-value are indicated in the table. Genes with p-value<0.001 in common in the three series are highlighted in bold.

	**GSE79196**		**EGAD00010001842**		**GSE93291**	
**Gene**	**R Pearson**	**P-value**	**R Pearson**	**P-value**	**R Pearson**	**P-value**
PXDN	0.57476	5.48E-8	0.68071	1.85E-7	0.19806	0.02875
GSTP1	0.68534	1.08E-6	0.68139	1.79E-8	-0.05429	0.55257
HBB	0.30459	0.0251	-0.07425	0.6532	-0.18278	0.04390
TP53INP1	0.13503	0.3303	0.15579	0.3436	-0.16401	0.07105
PRDX5	0.35100	0.0093	0.35862	0.0250	-0.11124	0.22254
TXNRD1	0.56030	1.05E-9	0.54317	0.0004	0.00165	0.98561
S100A9	0.15580	0.2606	-0.03440	0.8353	-0.27486	0.00218
MGST2	0.60113	1.54E-8	0.68495	1.51E-7	-0.02403	0.79278
TXNDC17	0.46540	0.0004	0.55906	0.0002	-0.18715	0.03901
PRDX1	0.39252	0.0033	0.48201	0.0019	0.08776	0.33646
PRDX3	0.31344	0.0210	0.41117	0.0093	-0.00254	0.97786
MGST1	0.16002	0.2477	0.25984	0.1102	-0.31357	0.00044

GPX7	0.22610	0.1002	0.33549	0.0368	-0.18082	0.04625
PTGS2	0.09925	0.4752	0.07758	0.6387	-0.17506	0.05377
GPX1	0.24505	0.0741	0.42576	0.0069	-0.27277	0.00237
CLIC2	0.37145	0.0057	0.49076	0.0015	-0.23026	0.01073
ALOX5AP	0.08838	0.5251	0.38961	0.0142	0.12943	0.15535
PRDX6	0.45065	0.0006	0.55083	0.0003	0.03805	0.67736
**PRDX2**	**0.50108**	**0.0001**	**0.53658**	**0.0004**	**0.29999**	**0.00079**
DHFR	0.29467	0.0305	0.32680	0.0423	-0.20387	0.02430
CD36	0.13585	0.3274	0.31038	0.0545	-0.34287	0.00011
FBLN5	0.32365	0.0170	0.55265	0.0003	0.08097	0.37529
ATP7A	0.20209	0.1428	0.56672	0.0002	0.12176	0.18156
PON2	0.86817	0.0019	0.82752	8..24E-3	0.29965	0.00080
PTPRK	0.36985	0.0059	0.11109	0.5008	-0.17368	0.05573
PKD2	0.66392	4.43E-6	0.65110	7.17E-8	0.17489	0.05402
NET1	0.44421	0.0008	0.58105	0.0001	0.20736	0.02192
**CD38**	**0.56894**	**7.16E-8**	**0.65526**	**5.98E-8**	**0.34477**	**0.00010**
MSRB2	0.62079	5.5E-7	0.65030	7.42E-8	0.13782	0.13008
APOD	0.37739	0.0049	0.40157	0.0113	0.02501	0.78451
DAPK1	0.33798	0.0124	0.45959	0.0032	-0.02585	0.77747

STAU2	0.45328	0.0006	0.53165	0.0005	0.21682	0.01645
ECT2	0.50016	0.0001	0.39742	0.0122	-0.29284	0.00106
PCNA	0.39088	0.0035	0.37323	0.0193	-0.28475	0.00148
CDK1	0.41111	0.0020	-0.38401	0.0158	-0.31530	0.00040
MELK	0.40846	0.0022	0.32052	0.0467	-0.14866	0.10223
TLR4	0.60173	1.49E-8	0.65675	5.6E-8	0.01221	0.89381
PAWR	0.48685	0.0002	0.36918	0.0207	0.36681	3.24E-9
EZH2	0.39720	0.0029	0.35374	0.0272	-0.03372	0.71233
MYEF2	0.20918	0.1290	0.29589	0.0674	0.27922	0.00184
**ABL1**	**0.64431**	**1.46E-7**	**0.54223**	**0.0004**	**0.53324**	**2.56E-4**
FOS	0.11240	0.4184	0.14462	0.3797	-0.09878	0.27903
PXN	0.47153	0.0003	0.67774	2.14E-8	0.26245	0.00350
SIRPA	0.36708	0.0063	0.73063	1.28E-7	0.17974	0.04759
ATOX1	0.39101	0.0035	0.55256	0.0003	-0.26959	0.00267
TBC1D24	0.37567	0.0051	0.35267	0.0277	0.28574	0.00142
NCOA7	0.32172	0.0177	0.47429	0.0023	0.11989	0.18839
TRPM2	0.55455	1.35E-9	0.50241	0.0011	0.04096	0.65422
STX2	0.27105	0.0474	0.29390	0.0694	-0.04312	0.63719
CASP3	0.27604	0.0433	0.47355	0.0023	-0.00644	0.94390

PPP2CB	0.45920	0.0005	0.72352	1.95E-7	0.28297	0.00159
HDAC2	0.40044	0.0027	0.33200	0.0389	-0.12237	0.17935
CHD6	0.47629	0.0003	0.20037	0.2213	0.13202	0.14719
AKR1C3	0.40729	0.0022	0.22018	0.1780	-0.07031	0.44155
NUDT1	0.28809	0.0346	0.49345	0.0014	0.13218	0.14668
IDH1	0.38574	0.0040	0.59712	5.99E-6	0.20413	0.02412
**ALOX5**	**0.49689**	**0.0001**	**0.58256**	**9.97E-9**	**0.48068**	**2.1E-6**
NDUFB4	0.26185	0.0558	0.49019	0.0015	0.30016	0.00078
PDCD10	0.47233	0.0003	0.33701	0.0359	-0.21797	0.01587
STAT1	0.20485	0.1373	0.36762	0.0213	-0.04320	0.63658
HSPB1	0.26701	0.0510	0.24577	0.1315	0.13916	0.12634
ALS2	0.43850	0.0009	0.30710	0.0572	0.07868	0.38901
ETV5	0.03560	0.7983	0.38486	0.0156	-0.23846	0.00817
KEAP1	0.31673	0.0196	0.36178	0.0236	-0.12555	0.16821
ANXA1	0.03949	0.7768	0.38204	0.0164	-0.08756	0.33754
C19orf12	0.21841	0.1126	0.33158	0.0392	0.24303	0.00699
BNIP3	0.24395	0.0755	0.44760	0.0043	0.07418	0.41678
RBX1	0.47266	0.0003	0.44740	0.0043	-0.14196	0.11882
ROMO1	0.27737	0.0423	0.56133	0.0002	-0.17122	0.05933

MCTP1	0.32083	0.0180	0.33088	0.0396	-0.18322	0.04338
AIF1	0.13007	0.3485	0.52965	0.0005	-0.22169	0.01413
HYAL2	0.41990	0.0016	0.48589	0.0017	-0.26038	0.00377
IMPACT	0.08578	0.5374	0.42547	0.0069	-0.29238	0.00108
MAPK8	0.16603	0.2302	0.28545	0.0782	0.22725	0.01183
ADAM9	0.02053	0.8829	0.28226	0.0817	-0.19814	0.02869
RBM11	0.27977	0.0405	0.07222	0.6622	-0.08997	0.32434
VKORC1L1	0.23504	0.0871	0.51996	0.0007	-0.18969	0.03638
SMPD3	0.25271	0.0652	0.43542	0.0056	0.16846	0.06362
PSMB5	0.31767	0.0192	0.51804	0.0007	0.11509	0.20681
CTNNB1	0.22164	0.1072	0.37637	0.0182	0.15978	0.07875
ERCC8	0.30364	0.0256	0.43482	0.0057	-0.09769	0.28443
APEX1	0.36581	0.0065	0.58990	7.744E-9	0.25438	0.00469
NDUFA12	0.23903	0.0817	0.28094	0.0832	-0.33401	0.00017

Table S3. List of primers used for RT-PCR

Gene	**Primers**
PRDX1	Forward: 5'-CCCTCTTGACTTCACCTTTGT-3'
	Reverse: 5'-TCCACAGAAGCACCAATCAC-3'
PRDX2	Forward: 5'-CAACGTGGATGACAGCAAGG-3'
	Reverse: 5'-AGGGCGTCACTATTCAGCTT-3'
PRDX3	Forward: 5’-GTAACATCTTGGTGGCTGGC-3’
	Reverse: 5’-AGGACCGTTGATCAGAAGCT-3’
PRDX5	Forward: 5’-CGGGTATGGGACTAGCTGG-3’
	Reverse: 5’-GGCTCTGCTGAAACTGCG-3’
PRDX6	Forward: 5’-GTCCTTCCAACCATCCCTGA-3’
	Reverse: 5’-AACACAATTGGCAGCTGACA-3’
VEGFA	Forward: 5´-CTACCTCCACCATGCCAAGTG-3´
	Reverse: 5´-TGCGCTGATAGACATCCATGA-3´
PDK1	Forward: 5´-GAAGCAGTTCCTGGACTTCG-3´
	Reverse: 5´-ACCAATTGAACGGATGGTGT-3´
GUSB	Forward: 5’-CGTGGTTGGAGAGCTCATTT-3’
	Reverse: 5’-GAACGCTGCACTTTTTGGTT-3’

SUPPLEMENTAL FIGURES

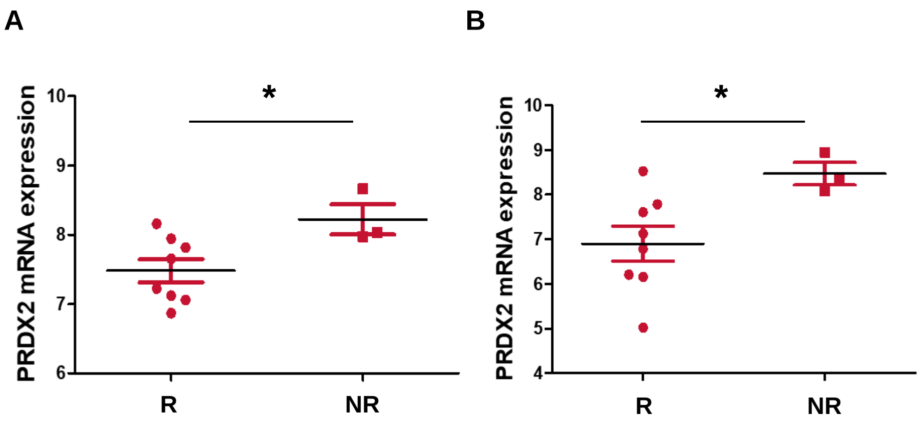

FIGURE S1. PRDX2 mRNA expression is higher in primary samples from non-responder than responder patients to chemotherapy. PRDX2 mRNA expression was analyzed in primary samples from responder (R) and non-responder (NR) MCL patients to chemotherapy, using two independent series of MCL cases, Series #1 **(A)** and Series # 2 **(B).** The significance of difference was determined by independent samples Student t test: *$p<0.05$.

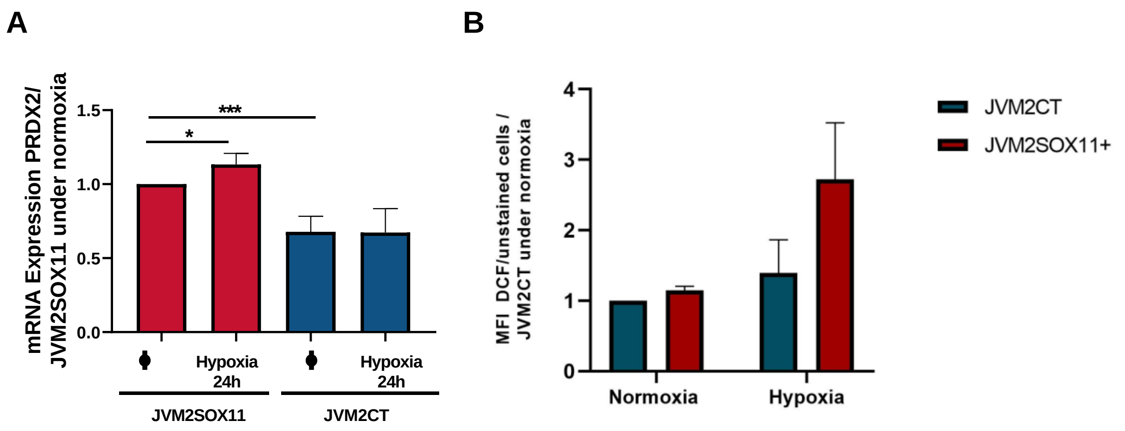

Figure S2. PRDX2 mRNA expression is upregulated by hypoxia upon extopical overexpression of SOX11 compared to control JVM cell lines. (A) PRDX2 mRNA expression levels, analyzed by qPCR, relative to GUS mRNA expression levels, in JVM2SOX11+ and JVM2CT MCL cell lines in normoxic (Ø) and hypoxic (1.2%O_2) conditions, for 24h. Results are refereed to JVM2SOX11+ cells in normoxia. **(B)** Median fluorescence intensity of DCF dye, in JVM2SOX11+ and JVM2CT MCL cell lines in normoxic (Ø) and hypoxic (1.2%O_2) conditions, for 24h. Results are refereed to JVM2SOX11+ cells in normoxia. The significance of difference was determined by independent samples Student t test: *p<0.05, **p<0.01.

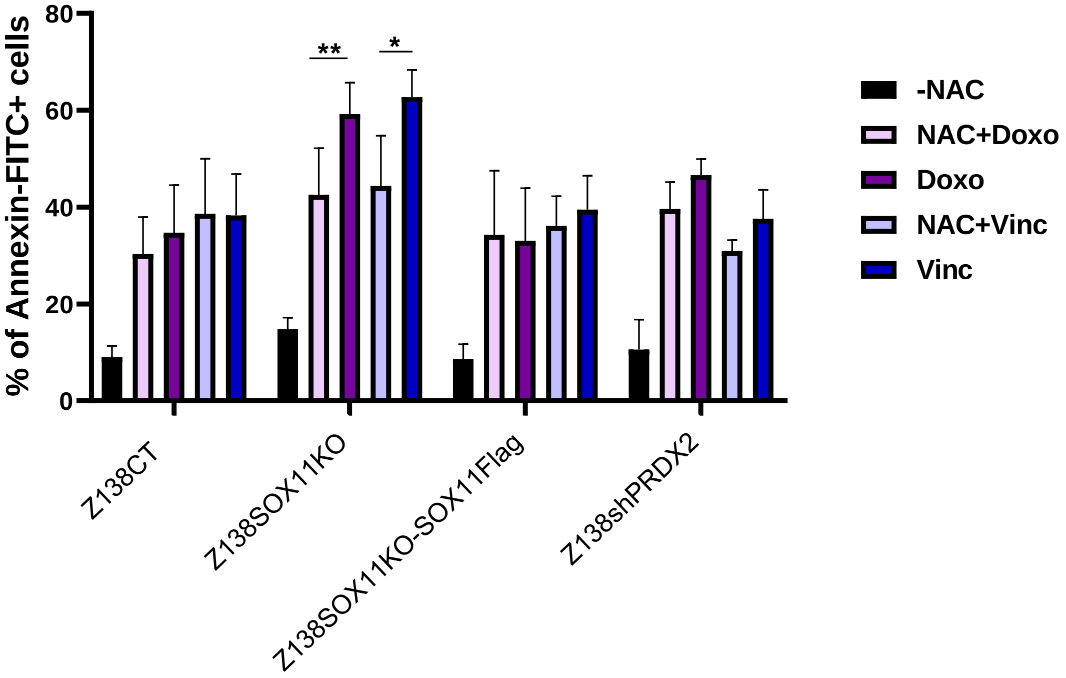

FIGURE S3. ROS depletion reduced cell death induced by drugs in MCL in absence of SOX11. % of Annexin-FITC+ in Z138wt, Z138CRISPRSOX11, Z138CRISPRSOX11-SOX11Flag and Z138shPRDX2 MCL cells after 0.05 µM doxorubicin (Doxo) and 0.01 µg/ml vincristine (Vinc) treatments in the presence of 2 mM N-acetyl-cysteine (NAC). Experiments were performed under hypoxia conditions (1.2%O_2 24h). The significance of difference was determined by independent samples Student t test: *p<0.05, **p<0.01.

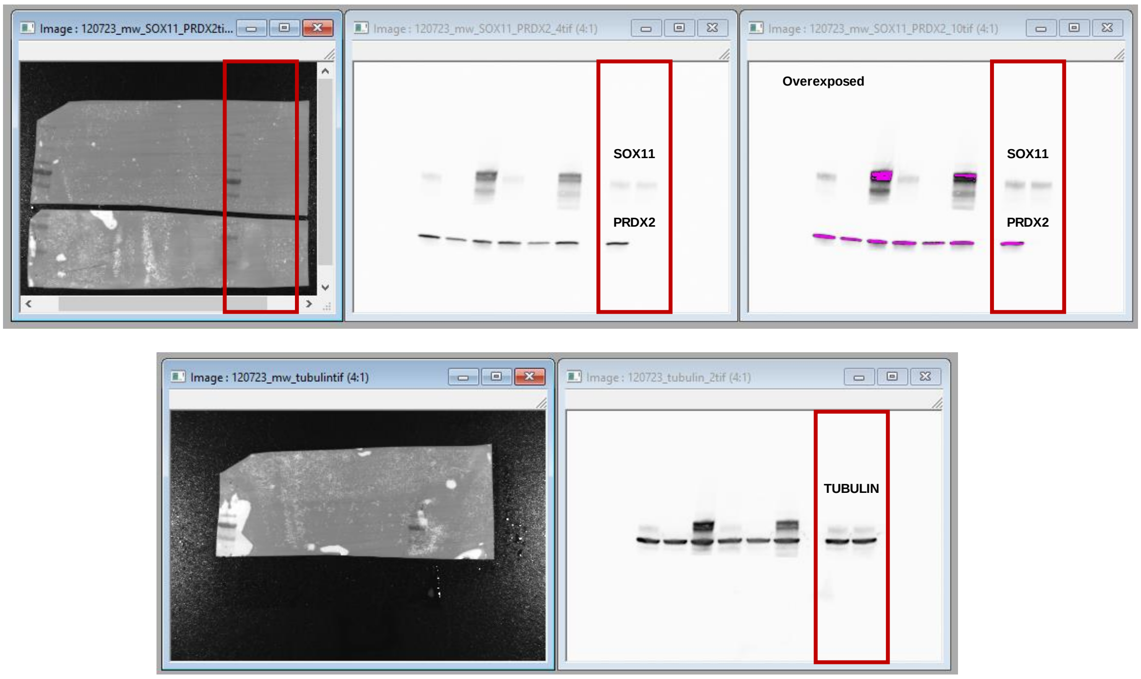

FIGURE S4. Full-length of the cropped blots grouped in Figure S4. We cannot provide the original images of full-length (FL) blots of Figure S4. The blots shown in Figures S4, were cut from a same FL membrane prior to hybridization with anti-SOX11, -PRDX2 and -Tubulin antibodies, as these proteins have different KDa and we would like to analyze then in same conditions of cells growing in same conditions, same western blot conditions, same conditions for hybridization. However, we are including images of all blots as they were cut from the same FL membrane and analyzed by WB. Membranes transferred with same protein extracts from Z-138CT and Z-138shPRDX2 cell lines and cropped to (**A**) blot with anti-SOX11 antibody (SOX11 protein; upper western blot) and anti-PRDX2 antibody (PRDX2 protein; bottom western blot). **(C-D). (C)** Cropped membrane transferred with same protein extracts as in membrane (A) from Z-138CT and Z-138shPRDX2 cell lines (**D**) and blot showing anti-Tubulin antibody (TUBULIN) as loading control.

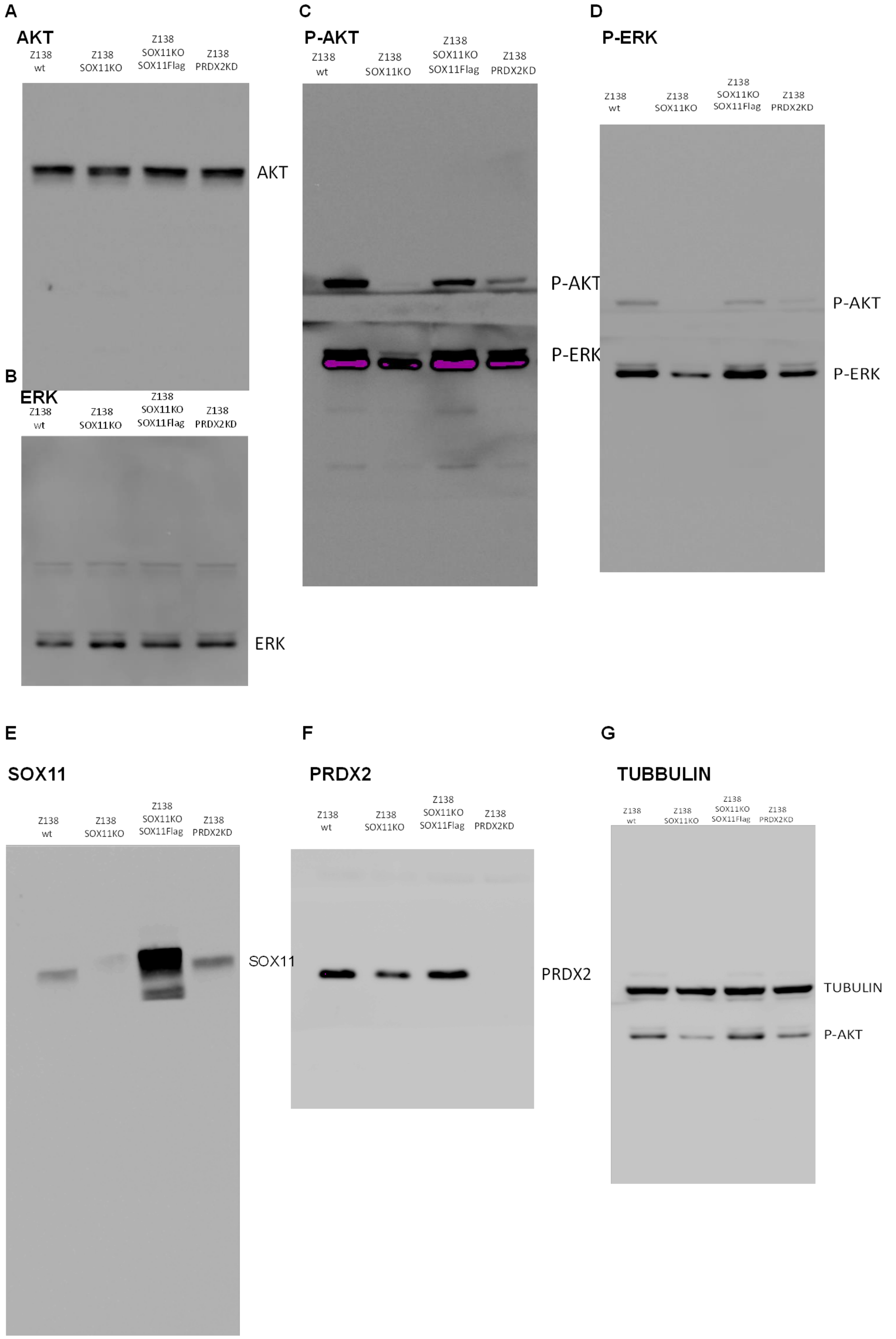

FIGURE S5. Full-length of the cropped blots grouped in Figure 5. We cannot provide the original images of full-length (FL) blots of Figure S5. The blots shown in

Figures S5, were cut from a same FL membrane prior to hybridization with different antibodies, as these proteins have different KDa and we would like to analyze then in same conditions of cells growing in same conditions, same western blot conditions, same conditions for hybridization…. However, we are including images of all blots as they were analyzed by WB, with membrane edges visible, for all hybridization in Figure S5. Membranes transferred with same protein extracts from Z138WT, Z138SOX11KO, Z138SOX11KOSOX11FLAG and Z-138PRDX2KD cell lines growing for 24h under hypoxia conditions (1.2%O_2) for all blots, and separated WB analysis showing **(A)** AKT, **(B)** ERK, **(C)** P-AKT, **(D)** P-ERK, **(E)** SOX11, **(F)** PRDX2 and **(G)** TUBULIN, used as loading control.
